# Papillary Mucinous Adenocarcinoma of the Endolymphatic Sac: A Rare Middle Ear Neoplasm

**DOI:** 10.7759/cureus.16413

**Published:** 2021-07-15

**Authors:** Muhammad Tahir, Cherish Frick, Ghassan Tranesh

**Affiliations:** 1 Pathology and Laboratory Medicine, University of South Alabama Hospital, Mobile, USA; 2 Pathology, Case Western Reserve University School of Medicine, Cleveland, USA; 3 Surgery, Lancaster General Hospital, Pennsylvania College of Health Science, Lancaster, USA; 4 Anatomical and Clinical Pathology, University of Arizona, Tucson, USA

**Keywords:** endolymphatic sac tumor, papillary mucinous adenocarcinoma, glandular neoplasm, middle ear tumor, neoplasm of temporal region and mastoids air cells

## Abstract

Glandular neoplasms of the temporal-mastoid region and endolymphatic sac (ELS) are rare, and it is quite challenging to differentiate between an adenoma and an adenocarcinoma. ELS tumors (ELST) usually present with papillary, follicular, or solid patterns and can be further distinguished histologically and through immunohistochemistry. The microscopic features and clinical course of this neoplasm have been comprehensively explained by Heffner, who considered it “low-grade adenocarcinoma of likely ELS origin.” The papillary form more commonly affects females, and it is a more aggressive form of ELST that is destructive and exhibits extensive local spread. The tumor usually has a close association with von Hippel-Lindau (VHL) disease, but 11%-30% of the ELST cases develop in individuals without a VHL mutation. ELSTs manifest with headaches, hearing loss, ear discharge, and cranial nerve palsies. Currently, the only available curative therapeutic intervention consists of wide local excision and long-term follow-up. Because of the sensitive location of this tumor, the adjuvant radiotherapy options are still questionable. In this case report, the author presents a 74-year-old woman with a past medical history of Schneiderian papilloma and was diagnosed with papillary mucinous adenocarcinoma of the ELS not associated with VHL disease.

## Introduction

The different parts of our body are derived from three different germ layers. Similarly, the membranous labyrinth and endolymphatic sac (ELS) are derived from the ectoderm and are found postero-medial to the temporal bone [1,2]. The endolymphatic duct connects with the proximal part of the ELS and the posterior segment of the ELS contiguous with the posterior cranial fossa [3]. The ELS is filled with endolymph, and its primary function is to monitor and control the volume, pressure, and proper flow of the endolymph. It also helps with hearing and equilibrium, and any change in the ELS homeostasis will manifest as symptoms of hearing loss, dizziness, tinnitus, and the development of Meniere's disease [4,5].

Middle ear tumors are sub-classified as adenomas, aggressive middle ear neoplasms, papillomas, and mixed or papillary type [6,7]. The mixed and adenomatous neoplasm subtypes are slow-growing and do not invade the surrounding structures. In contrast, the papillary neoplasm of the middle ear is a very aggressive tumor, and it can extend to the cranial cavity and mastoid bone, and often involves the cranial nerves and otic capsule [7-9]. In 1988, Gaffey et al. defined this middle ear tumor in the area of the ELS [8]. Subsequently, Gaffey and his colleagues revised nine identical cases specified in the literature and proposed the term “aggressive papillary middle ear tumor” [8]. Heffner identified similar tumors a year later and described them as low-grade papillary tumors of the middle ear originating from the ELS [10]. The nomenclature for this middle ear tumor has been reviewed by Mills et al., but ultimately, the World Health Organization (WHO) approved the broadly universal term “endolymphatic sac tumors” [11]. However, it must be remembered that the definitive development of this tumor from the ELS remains to be determined.

ELS tumors (ELSTs) are extremely uncommon, with only around 300 cases published in the literature [1]. The purpose of this case report is to discuss the histopathological presentation, diagnosis, and treatment of this rare entity.

## Case presentation

A 74-year-old female patient came in for a follow-up with an extensive past medical and surgical history. During the visit, her vital signs were as follows: blood pressure, 133/83 mmHg; heart rate, 88 beats/min; temperature, 97.5°F; respiratory rate, 16/min; and body mass index, 27.16. Her medications were the following: vitamin D, potassium, magnesium, aspirin 81 mg/day (calcium supplement), glucosamine capsule, and prednisone 20 mg as needed.

Past medical history: The patient has a past medical history of skin cancer, endometrial cancer, and Schneiderian papilloma of the right mastoid.

Past surgical history: The patient had undergone a bilateral hip replacement, hysterectomy, hemorrhoidectomy, breast lump resection, tubal ligation, resection of Schneiderian papilloma (June 2018), and excision of skin cancer from the right leg (December 2019).

In 2014, the patient presented with right-sided hearing loss. In 2017, her right tympanic membrane was perforated, with blood and abnormal tissue protruding from the eustachian tube orifice. Right ear tympanostomy tubes were placed, which helped. The tube eventually fell out, and the tympanic membrane healed. She had a recurrence of middle ear effusion, for which a computed tomography (CT) scan of the right temporal area was performed. The CT images with and without contrast showed complete opacification of the right middle ear cavity and mastoid air cells.

Because of the opacification of the middle ear and the mastoid air cells, an audiogram was recommended to test the extent of hearing loss. The results demonstrated mild sloping profound sensorineural hearing loss of the right ear with a conductive component at 2 kHz and mild sloping profound sensorineural hearing loss of the left ear. The pure tone averages were 60 dB in the right ear and 28 dB in the left ear. The word recognition scores were 36% on the right and 92% on the left. The fiber-optic examination revealed blood draining from the right eustachian tube orifice. The magnetic resonance imaging (MRI) results showed extensive fluid throughout the mastoid air cells and the middle ear with some relative enhancement.

She underwent right trans-temporal craniotomy and partial resection of the mass in January 2018. Histopathology was performed on the excised mass, which was reported as an ELST. In May 2018, a follow-up MRI revealed the recurrence of the lesion, and on physical examination, the lesion was protruding through the eardrum associated with otorrhea. In June 2018, a re-resection of the lesion was done, and this time, the eardrum, malleus, incus, and external canal bones were removed. There was a portion along the petrous bone and carotid artery where the tumor could not be removed, and the tumor extended from the middle fossa dura to the sigmoid sinus.

The patient then underwent radiation therapy (60 Gy in 30 fractions). In November 2019, the patient developed right facial palsy, which was treated with two weeks of prednisone without resolution. A follow-up MRI showed a new 5-mm nodule in the right nasopharynx. There was no radiographic reoccurrence of the tumor in the right temporal bone/fossa, and there was no evidence of facial nerve involvement.

In January 2020, the patient underwent a right-sided nasopharyngeal eustachian tube biopsy. The biopsy specimen was sent and processed in the pathology department, and the report showed a low-grade papillary neoplasm consistent with an ELST origin. The patient was scheduled for a follow-up MRI and CT in the next six months. The details about how the biopsy specimen was processed and the histopathological diagnostic features of ELST are discussed in the Methods and Materials sections below.

Methods and materials

The biopsy specimen was processed by fixing in formalin and embedding in paraffin before sectioning and fixing on glass slides. After the slides were processed and ready, they were stained with hematoxylin and eosin (H&E). In addition to H&E, some selected slides were also stained with periodic acid Schiff.

An immunohistochemistry (IHC) study was performed using the EnVision method. The process of immunostaining implies the selective recognition of antigens (proteins) in the tissue sections of cells by utilizing the fundamental rationale of antibody binding particularly to the antigens in the biological tissue. The antibodies that were used for IHC in this case of ELST were cytokeratin 7 (CK-7), pan-keratin, cytokeratin 20 (CK-20), cytokeratin 19 (CK-19), vimentin, glial fibrillary acid protein (GAF), thyroglobulin, and Ki-67. The slides were boiled under high pressure with citrate buffer (0.01 mole/L, pH 6.0) for the purpose of antigen retrieval. The proper positive and negative controls were also performed.

Histopathology

Low Power

**Figure 1:**
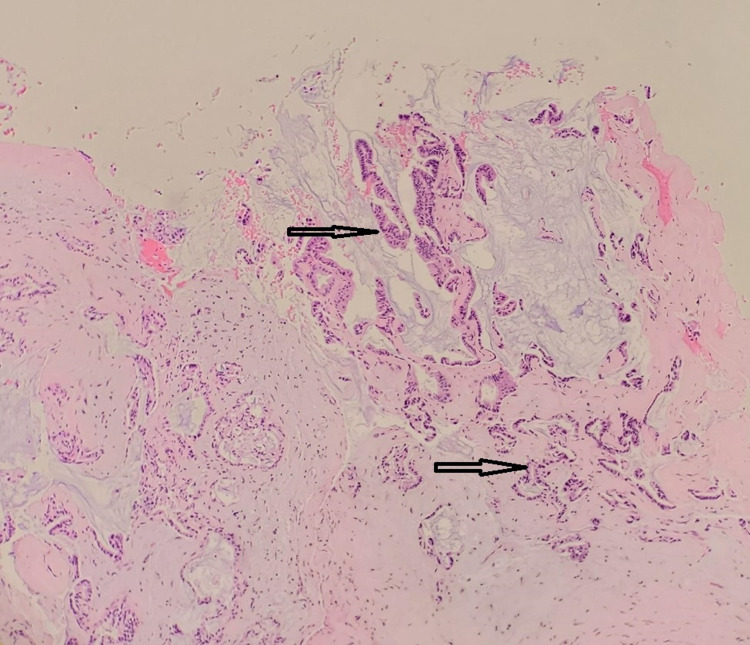
Papillary and focally glandular architecture

**Figure 2:**
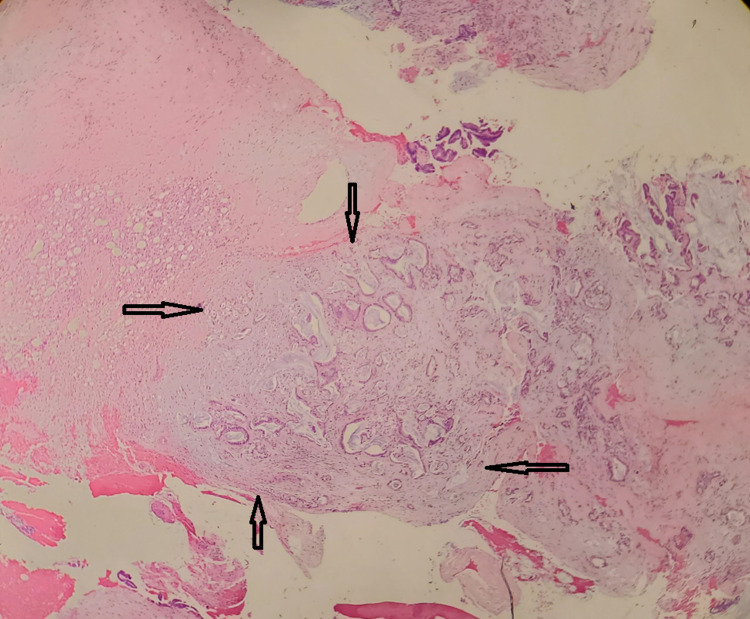
Neoplastic proliferation of tumor cells

^High Power^

Morphologically, at low power, the specimen showed mostly papillary and focally glandular architecture. The neoplastic proliferation of the tumor cells comprised columnar and focally cuboidal cells with round to oval nuclei that show mild to moderate pleomorphism with abundant extracytoplasmic mucin (Figures 1, 2). As mentioned above, the tumor cells were generally columnar or cuboidal and cohesive, often with distinct cell boundaries. Above the basement membrane and underneath the luminal cells was a layer of conspicuous myoepithelial cells. The epithelial cells have a clear-pale cytoplasm, luminal, central, and uniform nuclei. In the case of infected and old healing lesions, inflammatory infiltrates were often present next to neoplastic cells along with granulation tissue.

**Figure 3:**
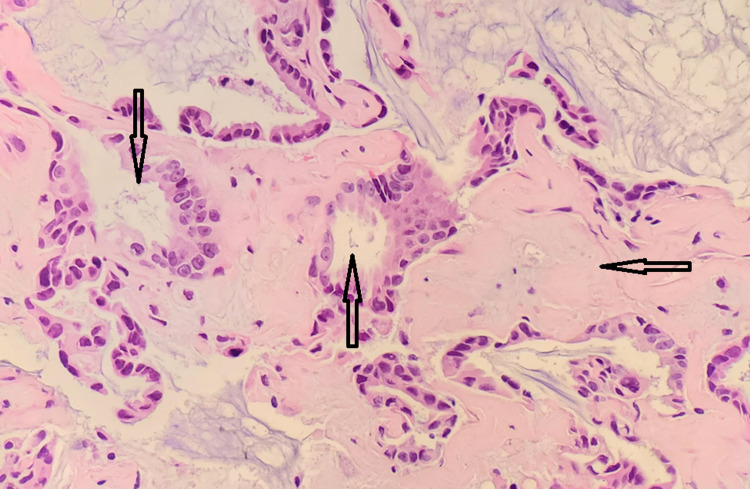
Multiple acinar configurations with an infiltrative growth pattern and desmoplastic response

^Immunochemistry^

On higher magnification, a single layer of columnar to cuboidal cells lined the papillary and glandular structures. Moderate to mild pleomorphism, rare mitotic activity, and necrosis were not observed. On the other hand, multiple acinar configurations with infiltrative growth patterns and desmoplastic responses were seen (Figure 3). Unconventional microscopic features that were not observed in our case of ELST but may be present in others include intermittent thyroid-like hypercellular regions with cystic glandular spaces filled with colloid substances, areas of hemorrhage, and necrosis with cholesterol clefts, mitotic figures, and structures that appear similar to choroid plexuses papillomas.

**Figure 4:**
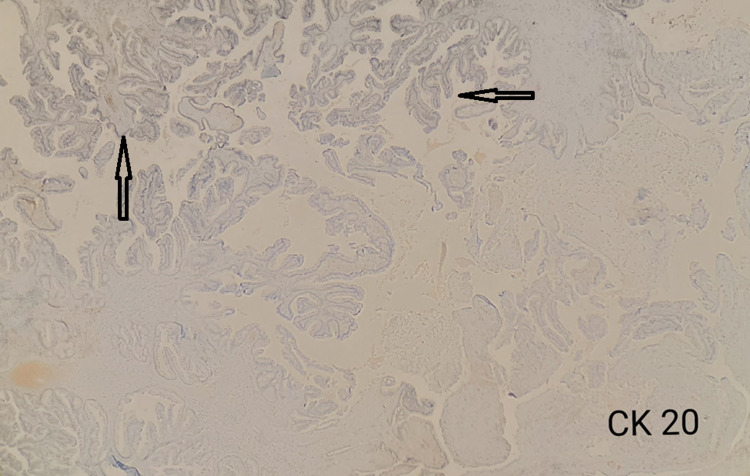
Negative CK-20, highlighting the papillary growth pattern CK, cytokeratin

**Figure 5:**
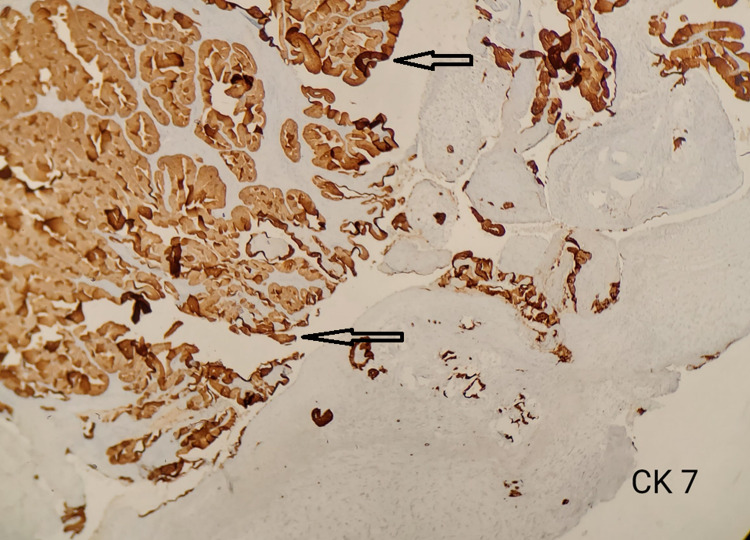
IHC staining showing positive immunohistochemical stain for CK-7 IHC, Immunohistochemistry; CK, cytokeratin.

^Diagnostic modalities for an ELST^

^Radiography^

As described in the Methods and Materials sections, an IHC study was performed. CK-20, which highlights papillary growth, was negative, whereas CK-7 immunostaining was positive as evident in Figures 4, 5.

In this case of ELST, both CT and MRI were used as the imaging diagnostic tools. The CT scan and MRI results showed opacifications in the middle ear and the mastoid air cells in 2018, during the initial presentation of the patient. After partial resection of the lesion, the patient developed a nasopharyngeal mass along with middle ear effusion, which was diagnosed through a CT scan and MRI in January 2020.

The CT scan and MRI techniques are widely used to diagnose ELST [12]. The CT scan images usually show a lytic temporal bone mass that typically extends into the posterior cranial cavity, which may present as a cerebellopontine angle tumor. In most cases, the tumor center rests at or near the temporal bone at the posterior medial surface [8,12].

^Angiography and Audiogram^

Apart from the imaging modalities, the most sophisticated procedure to diagnose an ELST in clinical practice is angiography. Compared to an angiography, an audiogram is a noninvasive test that was performed in this case of ELST. The audiogram showed that the patient had significant hearing loss in the right ear and moderate hearing loss in the left ear because of an invading ELST. A fiber-optic examination was also performed, which showed blood draining into the eustachian tube.

Angiography was not performed on this patient. However, according to previous angiography results in the literature, the tumor can have a dual blood supply from intra- and extra-cranial sources, making it extremely vascularized. A jugulotympanic paraganglioma is another entity that can mimic ELST because both of these tumors are highly vascularized and can carry potential bleeding risks [10].

^Differential Diagnosis of ELST^

Intrinsic temporal bone neoplasms are included in the differential diagnosis for ELSTs, with paragangliomas being the most common. Meanwhile, metastatic papillary thyroid carcinoma, metastatic renal cell carcinoma, and choroid plexus papilloma are histologically similar to ELSTs. The ELSTs are papillary cystic structures lined with a simple cuboidal or columnar epithelium and are heavily vascularized. Clear cells, as well as siderophages and cholesterol clefts, are visible (vacuolated cells). Nuclear pleomorphism is minimal, and mitoses are uncommon [13,14].

^Staging^

There are no universally accepted staging recommendations. Bambakidis et al. suggested the system described in Table 1 [12].

**Table 1:** Staging of endolymphatic sac tumor

Stage	Description
Stage I	Tumor limited to the temporal bone and middle ear cavity
Stage II	Tumor spreading to the posterior fossa
Stage III	Tumor spreading to the middle cranial fossa
Stage IV	Tumor spreading to the clivus and sphenoid wing

Schipper et al. defined type A tumors as those with no temporal bone destruction or dura invasion, type B as those with signs of osseous labyrinth infiltration and sensorineural hearing loss, and type C as those that have invaded the sigmoid sinus and jugular bulb [15,16].

The patient in this case underwent resection for a tumor that extended into the sigmoidal sinus and middle cranial fossa. If we follow the proposed staging system, then it is obvious that the tumor was an aggressive one, and the patient presented as stage III at the time of treatment.

^Treatment^

Complete resection with negative margins is the treatment of choice for ELST, but in advanced cases, this may not be possible. As the patient presented as ELST stage III, the only universally accepted treatment of choice is complete resection of the neoplastic tissue, which is recommended. Because of the complexity and advanced stage of the disease, the tumor was only partially resected. Under general anesthesia, she underwent a trans-temporal craniotomy and a partial resection of the lesion, along with resection of the eardrum, malleus, incus, and external canal bones. There were portions along the petrous bone, carotid artery, middle cranial fossa, and sigmoid sinus where the tumor could not be removed.

After the surgical resection, adjuvant radiotherapy and chemotherapy were administered with subsequent follow-ups. In January 2020, her follow-up MRI results showed a 5-cm lesion in the nasopharynx that was biopsied and confirmed as a reoccurrence of the ELST. The relapse of the ELST after resection confirms the aggressive nature and high recurrence rate of this condition.

Because the tumor relapsed, follow-up CT scans and MRIs were scheduled every six months, and close monitoring was recommended. From the patient's past medical and surgical history, she developed the ELST after the resection of the Schneiderian papilloma. Schneiderian papillomas are mostly benign, non-aggressive neoplasms that arise from the sinonasal mucosa. The development of ELST from a Schneiderian papilloma is quite rare. The pathological variants, classification, differentials diagnoses, etiological factors, clinical features, and treatment modalities of ELST and Schneiderian papillomas are elaborated in detail in the Discussion section.

## Discussion

Gaffey et al. labeled tumors with papillary architecture emanating from the middle ear epithelium and invading neighboring bony structures as “primary active papillary tumors of the middle ear” [8]. The complicated rugose part of the ELS, which stretches from its intraosseous base to its intradural extraosseous terminus, is thought to be the source of these tumors, according to Heffner's analysis of 20 cases [10]. Persons of both sexes aged 17-71 years are affected by the tumor. The therapeutic prodrome lasts for a long time, and hearing loss, otalgia, tinnitus, vertigo, and facial fatigue are the most frequent symptoms [7,12]. Examining the area behind the tympanic membrane reveals a red or blue tumor. An angiography will show that these tumors are hypervascular on a radiological level. Although these tumors do not metastasize, Bambakidis et al. demonstrated in a recent case study that they could spread to distant locations [12].

Centered on the clinical, radiologic, immunohistological, and electron microscopy observations, Heffner discovered a papillary neoplasm, most likely emerging from the ELS epithelium, which was a separate entity in 1989 [8]. The papillary tumor was characterized clinically and radiologically by the following: (1) locally aggressive growth with temporal bone destruction and, in most cases, a long clinical prodrome; (2) delayed detection of the tumor with extension to the posterior cranial fossa; (3) tumor core near the ELS, regardless of the size at the time of radiologic examination; and (4) 15% of cases are diagnosed with von Hippel-Lindau (VHL) disease, with the possibility of bilateral tumors [13].

One in every 39,000 individuals has VHL disease. According to current research, nearly one-third of all ELSTs are linked to VHL disease [14]. VHL is a hereditary disorder that is passed in an autosomal dominant pattern. Renal cell carcinomas, central nervous system and retinal hemangioblastomas, pheochromocytomas, and cysts of the kidneys, pancreas, and epididymis are examples of benign and malignant neoplasia.

Early routine audiology screening will enable early tumor identification. The likelihood of hearing restoration surgery should occur if there is a family history of VHL disease or, in the absence of an ELST, a diagnosis of VHL disease is made. Before surgery, detection of a tumor on gadolinium-enhanced MRI is required, followed by surgical exploration in patients with VHL disease and audiovestibular signs [15].

Hearing disturbances, vertigo, cerebellar disturbances, and facial nerve paralysis are some of the psychiatric manifestations of ELST. Almost half of the patients had hearing loss, and 44% of them had facial nerve paralysis and cerebellar abnormalities [10]. Papillary neoplasms resulting from the ELS epithelium were mostly overlooked before progressing to an advanced level. Giant tumors (>4 cm in diameter) that spread into the posterior cranial fossa and destroyed the temporal bone were discovered on radiologic inspection [13].

ELST is a papillary cystic neoplasm that appears similar to thyroid cancer on histology. Since the papillary part of the tumor is tiny, it is critical to separate it from middle ear adenomas, which typically do not have papillary architecture. Unlike paragangliomas, papillary tumors of the temporal bone are unmistakably epithelial. Papillary neoplasms resulting from the ELS and the choroid plexus have microscopic similarities, which may make diagnosis much more difficult. The neuroectodermal origins of the ELS and the choroid plexus are the same [17]. Both are present, as is endo-epithelial tissue from the mesothelium and thyroid epithelium, which line inner cavities and perform secretory and resorptive functions [18]. Tumors originating from the endo-epithelial tissues have a histologic appearance that is similar to papillary architecture. These tumors release cytokeratins, vimentin, and neuroendocrine markers in IHC. Transthyretin staining was suggested to differentiate between the ELS and choroid plexus tumors. This marker is more likely to be present in the choroid plexus epithelium and should be absent in ELST [19].

Heffner identified ELST as a low-grade adenocarcinoma in 1989 based on aggressive local development with bone damage, though metastasis has yet to be recorded [10]. Heffner coined the term “invasive papillary cystadenoma of endolymphatic sac origin” to describe these lesions. ELST is classified as an adenoma and adenocarcinoma of the middle and inner ear by the WHO [20]. Regardless of the tumor's biological origin, it is essential to remember that ELST has a locally disruptive behavior. Complete tumor removal is necessary, but in advanced stages, this may not be possible [13]. As a result, proper care entails detecting an ELST in its early stages based on the radiologic results and selecting a surgical procedure that allows the tumor to be removed.

Complete tumor resection is the treatment of choice. Only one patient with confirmed complete resection had a subsequent recurrence, indicating that complete resection of specific tumors is not possible without risking the devastating loss of function or death so that subtotal resection could be necessary. Patients who have had a subtotal resection may recover from postoperative radiotherapy. However, there is still a 50% chance of tumor regrowth, so careful monitoring is essential because re-resection may be needed. Subtotal resection accompanied by stereotactic radiotherapy has consistently resulted in tumor regrowth in published literature. Stereotactic radiotherapy has not demonstrated any advantage over standard fractionated radiotherapy in terms of survival or recurrence frequency [15].

Schneiderian papillomas

Glandular neoplasms of the head and neck are rare, and it is very confusing for clinicians to differentiate between the different subtypes. In our case report, the patient initially presented with a Schneiderian papilloma that was successfully resected. There is no established relationship between Schneiderian papillomas and ELSTs in the literature [21].

The nasal mucosa is lined by pseudostratified ciliated columnar epithelium, also known as the respiratory epithelium, and is derived from the ectoderm. The respiratory epithelium lines the sinonasal tract and is also known as the Schneiderian membrane, which is a source of three histologically and structurally different types of papillomas: inverted, fungiform, and oncocytic papillomas. In turn, these are collectively known as Schneiderian papillomas [22].

The classification, pathology, taxonomy, and etiology of sinonasal tract papillomas are disputed. In 1854, Ward et al. described these papillomas for the first time, and over several years, more than 50 various terminologies have been used to describe these clinical entities [22]. Almost 100 years later, in 1971, Hyams et al. made a significant effort to reclassify these tumors based on their histopathological and clinical characteristics. He classified these tumors into three main subtypes; fungiform, inverted, and oncocytic papillomas [23].

All three subtypes are collectively called Schneiderian papillomas and are very rare, occurring in only 0.4%-4.7% of all sinonasal tract tumors. [24]. Nasal polys are 25-60 times more common than Schneiderian papillomas [25].

Although there is uncertainty in the etiology of these papillomas, the inverted and fungiform subtypes have been suggested to be viral in origin. The different occupational and environmental factors, toxic agents, smoking, injury, and inflammation may affect the development of these lesions, but these are not very potent factors [8,25].

Fu et al. have reported nine different cases where patients with Schneiderian papillomas also had a history of anogenital papillomas. However, another study found that patients with sinonasal tract papillomas do not have an increased risk of growing papillomas in other sites of the body [26].

The prevalence of the different types of Schneiderian papilloma changes according to the institution. Fu et al. (1992) jointly reviewed 728 cases of Schneiderian papillomas. They reported that among the different types of papillomas, 6% were oncocytic (range, 2%-26%), 32% were fungiform (6%-50%), and 62% were inverted (47%-78%) [26-28].

Types of Schneiderina papilloma

(1) *Oncocytic Schneiderian Papilloma (OSP)*

Among the three different forms of Schneiderian papillomas, the OSP is very uncommon, and it is morphologically similar to the inverted variant. Histologically, these two entities are different and do not share features [29]. The most common presenting symptoms of OSP are nasal obstruction, infection, and occasional epistaxis. They tend to occur mainly on the lateral nasal wall or in the ethmoidal or maxillary sinuses. An OSP appears as a red-brown, fleshy pink, or grayish-looking papillary or polyploidy mass on gross pathological examination. Histologically, OSPs can be further divided into two categories: the cylindrical cell and columnar cell papillomas [30,31].

Etiology

Etiologically, an OSP is not associated with any viral infection. Many of the patients diagnosed with OSPs have tested negative for the human papilloma (HPV) virus through a polymerase chain reaction. This is in contrast with the other two subtypes of Schneiderian papillomas, the inverted and fungiform types, which show a close association with a viral infection. The most commonly affected age group is the middle-aged population, and this lesion is uniformly distributed between males and females [32].

Histopathology

Microscopically, the OSP epithelium is comprised of several layers of tall columnar cells, mostly two to eight cells in a layer. The cells have a slightly granular cytoplasm, which is suggestive of oncocytes. Barnes et al. have suggested and proven that the cells show similar characteristics with that of oncocytes but harbor abundant mitochondria and have very high amounts of cytochrome C oxidase content, thereby clearly attaining their oncocytic characteristics [29]. The nuclei are hyperchromatic, uniform, and somewhat vesicular, with hardly visible nucleoli. The nasal mucosa is always lined by ciliated pseudostratified columnar epithelium. In this OSP, the cilia are usually absent and can be identifiable in the different growth stages in a few cells.

(2) *Fungiform Papilloma*

FPs usually affect males between 20 and 50 years of age and are almost 10 times more prevalent in males. On gross examination, FPs are papillary, exophytic, or warty types of lesions that are usually gray, pink, or tan. They typically grow on the anterior part of the nasal septum and compared with OSP, they do not involve the paranasal sinuses [33]. FPs are rarely bilateral. Usually, they are multifocal, isolated, and distinct lesions. Patients with FPs can present with symptomatic growth, but unilateral or bilateral nasal obstruction and epistaxis are the most usual clinical presentations [34,35].

MRI and CT are the radiological tools used to diagnose Schneiderian papillomas, but the imaging results of FPs are usually not significant. Etiologically, it was suggested that the FPs are caused by HPV, particularly by types 6 and 11 and infrequently by types 16 and 57b. A total of 79 cases of FP were evaluated by polymerase chain reaction for the presence of HPV, and 45 cases (57%) were positive [36,37].

FPs should be differentiated from other very common keratinizing appendiceal papillomas such as warts and verruca Vulgaris near the nasal cavity. The presence of ciliated pseudostratified columnar epithelium with less keratinization can help diagnose and distinguish FPs from appendiceal papillomas [21].

The treatment of choice for FPs is complete surgical resection. The local recurrence of FPs is 22%-50%, which has been reported in the literature, and the most common cause of recurrence is incomplete surgical excision [33].

Histopathology

On gross examination, FPs can grow from a few millimeters to up to 2-3 cm and are divided into three subtypes: squamous, septal, and exophytic papillomas. Microscopically, the FPs are composed of a central core of fibro-vascular tissue covered by ciliated pseudostratified columnar epithelium roughly 8-20 cells thick. Commonly the FPs originate from the inner nasal mucosa, which is why the epithelium lacks keratinization properties. In some rare instances, if inflammation, infection, or trauma to the nose or the papilloma is too severe and causes the lesion to push out of the nose, it can be dried by air and may attain keratinization characteristics [8,21,22]. Most of the articles in the literature have reported that FPs are not associated with invasive squamous cell carcinomas. Nevertheless, Norris [33] and Buchwald et al. [35] have each declared a rare incidence of the coexistence of an FP and invasive squamous cell carcinoma.

(3)^ ^*Inverted Papilloma (IP)*

The third and last type of Schneiderian papilloma is an IP. This differs from OSP and FP in many aspects, but they all share a common site of origin, which is the sinonasal tract mucosa. IPs are classified based on their histological characteristics, and they account for 1%-4% of all sinonasal tract malignancies [38,39]. The prevalence of IP is higher in males than in females, accounting for a 3.4:1 male: female ratio as described by Lawson et al. [40].

Pathology

On gross examination, IPs appear as a polypoid mass that is bulky, soft, mildly firm, and granular and are pink to brownish to gray. The most frequent site of origin is the sinonasal tract, specifically the lateral nasal wall and septum. The involvement of the ELS, lacrimal system, paranasal sinuses, and nasopharynx is rare, but they have been reported as sites of origin [40,41].

Sign and Symptoms

The clinical presentation of IP is pretty similar to the other types of Schneiderian papillomas. Patients usually present with nasal obstruction that can be unilateral or bilateral, increased sinus pressure or infection, facial pain and pressure, headache, rhinorrhea, and epistaxis [41,42]. Apart from the clinical symptomology, imaging techniques such as CT and MRI are also helpful in arriving at a diagnosis, and they may show a soft tissue mass with little or no bony erosion.

*Etiology*​​​​

The definitive etiology of IPs is anonymous. Similarly, to FP, IPs are also associated with HPV infection. Remarkably, HPV strains 6, 11, 16, and 18, and rarely HPV 57. The Epstein-Barr virus (EBV) is also thought to be involved in the pathogenesis of IPs. Macdonald et al. have reported 13 out of 20 cases of IPs that were associated with EBV and confirmed by PCR [38]. Indeed, more research and data are needed to prove the definitive relationship between HPV and EBV as the causative agents of IPs [39].

Histopathology

Histologically, IPs are commonly nonmalignant lesions and rarely complicated by carcinomas, especially squamous cell carcinomas, mucoepidermoid, spindle and clear cell carcinomas, and warts [40,43]. They appear as an inverted growth of epithelial cells invading into the rudimentary submucosa and stroma on low power under the microscope. The epithelium is comprised of non-keratinized ciliated columnar cells that are usually 10 to 30 cells thick combined with goblet cells [38]. An IP should be treated aggressively by surgical excision because it tends to recur and transform into a malignancy. Endoscopic resection is preferred over lateral rhinotomy and medical maxillectomy because of the low recurrence rate and favorable prognosis [43,44].

## Conclusions

ELST is a unique neoplasm that originates from the ELS epithelium and can be malignant or benign. Malignant lesions exhibit an aggressive nature, with local destruction of tissues but a low risk of distant metastasis. Early wide surgical excision with negative margins is recommended for small lesions and has been the treatment of choice for many years. Because of the structural ambiguity of the ELS and distinct tumor territorial expansion patterns, complete local excision for massive tumors becomes highly challenging. Alternatively, in advanced cases where lesions invade the neighboring structures, radiotherapy, and gamma knife surgery may be the only options.

Almost 20% of ELSTs are associated with VHL disease, but there was no VHL mutation in our case. The patient had a history of Schneiderian papilloma two years ago, which then developed into papillary mucinous adenocarcinoma of the ELS. A Schneiderian papilloma is a benign tumor arising from the nasal mucosa and has very low malignant potential and local spread. The development of papillary mucinous adenocarcinoma of the ELS in a patient with a history of Schneiderian papilloma is also sporadic. More cases and studies are needed to prove the association between Schneiderian papillomas and ELSTs.

The case was identified because of its unusual presentation. Patients with head and neck neoplasms should be histopathologically diagnosed at an early stage for them to receive early treatment and follow-up. More research needs to be done to better understand the pathological and molecular mechanisms involved in the malignant transformation of ELST and the relationship between Schneiderian papillomas and papillary mucinous adenocarcinomas of the ELS.
